# Lightning Rods, Earthquakes, and Regional Identities: Towards a Multi‐Scale Framework of Assessing Fracking Risk Perception

**DOI:** 10.1111/risa.13167

**Published:** 2018-08-15

**Authors:** James A. Pollard, David C. Rose

**Affiliations:** ^1^ Department of Geography University of Cambridge Cambridge UK; ^2^ School of Environmental Sciences University of East Anglia Norwich UK

**Keywords:** Hydraulic fracturing, risk perception, social license to operate

## Abstract

Hydraulic fracturing has provided a persistent, polarizing, and highly politicized source of controversy internationally and in numerous national contexts for just under a decade. This research uses hydraulic fracturing (i.e., fracking) operations in New Zealand as a vignette through which to understand the underlying causes of controversy and the appropriateness of attempts to address them. A multi‐method approach using interviews (*n* = 25), diagrammatic analysis, and newsprint media was applied to evidence two major findings. First, previous attempts to explain fracking controversy based on social constructivist theory lack a multi‐scalar approach to the assessment of factors that influence risk perceptions. It is found that risk perception surrounding fracking in New Zealand reflects intra‐scalar interactions between factors originating at the international, national, regional, and local scale. Second, there is a concerning absence of critique pertaining to the concept of “social license to operate” (SLO), which has been advocated both internationally and nationally as an appropriate form of stakeholder engagement. This article contributes to the SLO outcomes literature by establishing a need to consider multi‐scalar influences on risk perception when explaining diverse SLO outcomes in communities where fracking operations are prospective or already taking place.

## 1. INTRODUCTION

Guaranteeing secure, sustainable, and affordable energy is one of the central challenges faced by modern societies. As history attests, onshore energy development often goes hand‐in‐hand with intense public opposition (Devine‐Wright, 2009; Flynn, 2003; Pidgeon & Demski, 2012). Hydraulic fracturing (henceforth referred to as fracking) has been applied in the oil and gas industry since 1949 to improve the productivity of existing wells and allow the extraction of unconventional resources such as shale gas (Montgomery & Smith, 2010; The Royal Society & Royal Academy of Engineering, 2012). It involves pumping “fracking fluid” down a well at high pressure to fracture source rock. So called “proppants,” typically spherical sand, in the fluid ensure fractures remain open after the fluid is removed, allowing gas to flow from the source rock via a multiple lined well to the surface.

A contentious, polarizing, and highly public debate has been playing out since 2010 between industry, regulators, scientists, activists, politicians, and the wider public over the technique popularly termed “fracking” (Mazur, 2014). Eight years on there remains no consensus as to whether fracking is the key to a “golden age of gas,” or a technology compounded by uncertain social and environmental impacts (International Energy Agency, 2011). The lack of consensus is manifest in an inconsistent array of policy responses ranging from pro‐fracking legislation (Pennsylvania), to moratoria (the Netherlands, Quebec, and Scotland) and complete bans (France).

Since fracking is a relatively new arena of energy controversy on the world stage, the literature on understanding people's risk perception towards the technique is undeveloped as compared to other energy projects, such as nuclear power. Using a vignette of fracking in New Zealand, the purpose of this article is twofold; first, it argues that a comprehensive multi‐scale approach (considering spatial scales from local to international) of assessing risk perception surrounding fracking has been largely missing from existing studies. This is a significant shortcoming considering the multi‐scale influences on fracking risk perception.

Second, the article extends existing work on “social license to operate” (SLO) (Gunningham, Kagan, & Thornton, 2004) to fracking, a context to which it has seldom been applied in the literature. Using the same vignette, the article argues that explicit attention to public risk perception grants greater sophistication to existing explanations of SLO outcomes.

## 2. THE RISKS OF FRACKING: A CONSTRUCTIVIST APPROACH

### 2.1. Understanding Risk Perception

Although risk has historically been defined in objectivist terms, scholars have illustrated the complexity of risk perception (Beck, 1992; Slovic, 1987; Walport & Craig, 2014). The “objectivist,” technical interpretation puts forward a quantitative definition of risk as probability multiplied by consequence. This approach has proven successful in characterizing risks in well‐defined systems where probabilities and consequences can be easily identified and quantified; e.g., the chances of engineering failure or transport safety (Stirling, 2007; Wynne, 1992). Yet, an insistence on applying these techniques to increasingly complex risks has revealed considerable limitations, with technocratic approaches to risk assessment criticized for creating an unsupportable sense of certainty (Wynne, 1992); purporting a misconceived separation of scientific investigation from its social and cultural context (Jasanoff, 1991); and failing to engage with the political implications of these risk assessments (Owens, 2000).

The unsatisfactory nature of objectivist interpretations has given rise to a collection of alternative approaches that fall into the broad category of constructivist theory. Constructivist approaches have displayed explanatory power in relation to numerous emerging techno‐environmental risks, including: nuclear power (Flynn, 2003), nanotechnology (Kahan, Braman, Slovic, Gastil, & Cohen, 2009), and renewable energy (Devine‐Wright, 2009). Suggesting a potential to offer significant insights to the controversy surrounding fracking. A constructivist approach explains mismatches between risk perception and quantitative risk assessment by drawing attention to a variety of less tangible cultural, psychometric, and place‐based factors. Cultural theory suggests that even if it were possible to determine risk quantitatively, the extent to which this risk can be considered “acceptable” will depend fundamentally on the value sets, or “worldviews,” of the communities from which the risk is perceived (Douglas, 1992, 1966). In this way, worldviews act as “orientating dispositions” that influence the uptake and interpretation of information concerning specific risks (Dake, 1992; Starr, 1969). Alternatively, and paying greater attention to the individual, psychometric theory provides a basis for explaining and anticipating public perceptions of risk, based on qualitative risk characteristics and emotive associations it acquires (Fischhoff, Slovic, Lichtenstein, Read, & Combs, 1978; Sandman, 1987; Slovic, 1987; Starr, 1969).

An area of research with purchase among practitioners is the study of socioeconomic and place‐based factors (Larock & Baxter, 2013). This includes, though is not limited to, opposition arising in the local proximity to development projects, collectively referred to as NIMBYism (Dear, 1992). It has been found that proximity alone is insufficient to explain opposition to local developments (Cotton, 2013; Severson, 2012). Rather, it is necessary to take into account ways in which a certain place contributes to the identity of communities and the specific associations the community has with the threat in question (Larock & Baxter, 2013; Perlaviciute & Steg, 2014; Vorkinn & Riese, 2001).

One attempt to reconcile these influences on risk perception is the social amplification of risk framework (SARF) (Kasperson et al., 1988). This is underpinned by the fact that as humans observe and communicate risk, they introduce subjectivities (Kasperson et al., 1988). Specific events associated with a given risk can be termed “risk events” and their “risk signal” can be analyzed as it becomes subject to processes of social amplification and attenuation (Kasperson, Kasperson, Pidgeon, & Slovic, 2003). It may even be possible to identify amplification or attenuation “stations,” such as institutions, people, or media sources, that act as sites of risk signal alteration. Following the amplification and attenuation process, the risk signal may exert ripple effects, leading to impacts on secondary or tertiary parties that were previously uninvolved (Kasperson et al., 2003).

### 2.2. Fracking Risk Perception—A Multi‐Scale Approach

Fracking can be considered an “emerging technology” given its recent rapid growth accompanied by significant uncertainty and ambiguity surrounding environmental impacts (Halaweh, 2013; Rotolo, Hicks, & Martin, 2015). Despite numerous publications (Moniz, Jacoby, & Meggs, 2011; The Royal Society & Royal Academy of Engineering, 2012) seeking to quantify the environmental and health risks associated with fracking, controversy persists at multiple scales from the local to the international. Few studies have turned to constructivist theory to explain the enduring controversy surrounding fracking operations, though the potential for it to provide the theoretical basis for analyzing perceptions of fracking has been outlined (Boudet et al., 2014; Clarke et al., 2015).

Existing literature has demonstrated the importance of different scalar influences on fracking risk perception. The individual is the most common scale of analysis. Research in the United States used a nationally representative survey (*n* = 1,061) to draw out key traits that correspond with support or opposition to fracking (Boudet et al., 2014). By considering sociodemographics, affective imagery, proximity, worldviews, political ideology, media use, and familiarity, it is suggested that women, those with egalitarian worldviews, and those who read the newspaper more than once a week are more likely to oppose. Drawing on the same data set, a later study sought to emphasize the importance of community identity in influencing risk perceptions, alluding to the importance of multi‐scalar influences on risk perception (Boudet, Bugden, Zanocco, & Maibach, 2016). A further study (Davis & Fisk, 2014) suggested that preferences regarding fracking regulation relate to whether people consider fracking as an energy (more positive) or an environmental (more negative) issue. A similar methodological approach was applied to focus explicitly on the role of top‐of‐mind associations, suggesting that “fracking” elicits more negative associations as opposed to “shale oil or gas development” (Clarke et al., 2015). By comparison, a study focusing on engagement with specific documentaries found that narratives may trigger affective responses in individuals (Cooper & Nisbet, 2016). Narrative involvement with the *Gasland* documentary increased worry, concern, and desire for regulation, while narrative involvement with the *FrackNation* documentary had a reassuring effect and engendered confidence in existing regulation.

One of the few studies (Graham, Rupp, & Schenk, 2015) to consider regional and community scales utilized 66 surveys in the United States to explore state‐dependent differences in risk perception. When choosing between increased regulation or a ban, at the national scale, the majority of respondents favored regulation, while in New York State, the majority favored a ban. By drawing on multiple surveys, each situated at specific scale, it is not possible to determine how multi‐scalar influences are reflected in any one person's or group's perception. To achieve this, multi‐scalar analysis of a single interview group is required.

At the national scale, media representations of fracking have been used to provide comparison between Poland, Germany, and the United Kingdom (Upham, Lis, Riesch, & Stankiewicz, 2015). Here, the multi‐scalar perspective refers to levels of socio‐technical change, ranging from niche‐protected innovations to landscape‐scale change. A further study (Sica, 2015) seeks to explain why fracking proceeded in Pennsylvania despite widespread opposition and rejection elsewhere nationally. The paper considers fracking advertisements, noting that benefits were convincingly presented at multiple stacked scales (local, regional, national), but the costs at each scale were not comprehensively recognized. While this study draws on multiple scales to explain national‐level policy differences, it does so only regarding the communication of cost and benefit, rather than multi‐scale influences on risk perception.

At the international scale, and with reference to both *Gasland* and the Maconodo oil spill, it has been demonstrated through news trend analysis in the United States, United Kingdom, and Australia that such signals have the potential to influence perceptions beyond the national context in which they were produced (Mazur, 2014). A social and mass media analysis in the United States found that the *Gasland* documentary both created a discursive opportunity for the fracking debate, and influenced the selection of issues that gained most attention (Vasi, Walker, Johnson, & Tan, 2015). The influence of these major news stories at finer scales remains to be determined.

Thus far, few studies provide a comprehensive consideration of the multi‐scalar nature of risk perception in the context of fracking. This is a significant omission because by situating analysis at a single scale, influences outside the scale of interest are side‐lined, and thus the fullest possible appreciation of reasons for opposition to (or indeed support of) fracking cannot be attained. Extending existing scholarship, this study addresses risk perception through a multi‐scale analysis of fracking operations with attention to local, regional, national, and international context.

### 2.3. Social License to Operate

While a multi‐scale analysis of risk perception surrounding fracking is important for critical scholarship, it is further essential to inform the practical assessment and management of fracking operations. Reliance on the objective risk definition has typically encouraged technocratic risk assessment, which often fosters a deficit model of stakeholder interaction. This is guided by the reasoning that if stakeholders can be made to understand the technicalities of a given risk, they will be more accepting of the implementation of technology associated with it. This assumes that differences in the assessment of risk between stakeholder groups can be attributed to differences in knowledge about the risk in question (Eden, 1998). Despite increasing attempts to engage the public, motivated by a desire to avoid public backlash, there are many instances where an unconvinced public has strongly rejected reassurances from regulators and industry based on technocratic risk assessments (Kahan et al., 2009; Pidgeon & Demski, 2012; Wynne, 1992).

The need to incorporate a broader range of stakeholders is encompassed in the concept of “social license to operate” (SLO), which refers to the idea that operators must go beyond compliance, making an effort to engage actively with the communities in which their operations are based (Gunningham et al., 2004). The conditions of the social license are achieved when the operator is judged as “having the ongoing approval and broad acceptance of society to conduct its activities” (Prno & Slocombe, 2012: p. 346). Such a license purportedly grants communities a more privileged position in the policy‐making process, representing a shift in governing authority towards the impacted communities (Prno & Slocombe, 2012).

Although the term itself emerged in the 1990s, literature commenting on the conceptual and practical suitability of the SLO concept is more recent (Boutilier & Thomson, 2011; Owen & Kemp, 2013; Prno, 2013; Prno & Slocombe, 2014). Research utilizing mining industry case studies has established: the novel contribution that SLO makes to stakeholder engagement (Boutilier, 2014); the relationship to sustainable development (Owen & Kemp, 2013); the challenge of implementing and measuring SLO in practice (Boutilier & Thomson, 2011); and how the managers of extractive industries themselves conceive of the concept (Parsons, Lacey, & Moffat, 2014). This last study found the majority of 16 Australia mining managers interviewed conceived of SLO as applying only locally. This viewpoint fosters conflictual relationships between companies and communities, with a suggested way forward being for company behavior to align with wider cultural values and to incorporate societal and local concerns (Parsons et al., 2014).

This recommendation is consistent with studies that show how SLO outcomes depend on context, public participation, trust, and culture (Harvey & Bice, 2014; Moffat & Zhang, 2014; Prno, 2013; Ruckstuhl, Thompson‐Fawcett, & Rae, 2014). One study incorporates these influences in a systems‐based conceptual framework for assessing the determinants of SLO outcomes (Prno & Slocombe, 2014). The framework emphasizes the need to engage with local variables specific to the community and mining project, alongside socioeconomic conditions, biophysical conditions, and governance/institutional arrangements that take place at regional, national, and international scales. The framework recognizes that local perceptions are an important influence on SLO outcomes. It seems likely, therefore, that outcomes may depend on the perception of risk associated with the activity in question. In theory, communities can reserve their “social license” if they perceive the risks associated with an activity, whether tangible or intangible, to outweigh the associated benefits.

The lack of critical commentary on SLO in contexts beyond mining is concerning. Fracking is an important context for analysis given the SLO concept is named as the ultimate goal of the International Energy Agency's “10 Golden Rules for a Golden Age of Gas” (International Energy Agency, 2011). Few studies assess the application of the SLO approach to fracking operations. One U.S. study offers guidance on how best to achieve SLO for fracking operations, though focusing explicitly on the need for best practice in attenuating local impacts on air, water, “nuisances,” monitoring, and disclosure (House, 2013). Similarly, a study making recommendations for an emerging fracking industry in China outlines local community impacts that must be addressed to achieve SLO (Hu & Xu, 2013). Both studies assume that failure to achieve SLO is a result of failure to address (largely) tangible impacts at the local scale. Further research is required to establish whether risk perception is subject to similar scalar interconnectivity as other factors in Prno and Slocombe's (2014) conceptual framework and whether this can contribute to understanding varied SLO outcomes.

## 3. METHODS

This study uses the case of fracking in New Zealand as a vignette to illustrate the need for a multi‐scale approach to understand risk perception, as well as extending the critical literature on “social license to operate.” Despite the small scale of current operations, New Zealand has not escaped the global controversy surrounding fracking. The technique was first applied there in 1989, in Taranaki, on the North Island (Parliamentary Commissioner for the Environment, 2012). Between 1989 and 2012, 55 wells were fractured, some, multiple times. Most operations targeted conventional “tight sands” in Taranaki, though some have taken place in coal seam gas mines in Southland and Waikato (Parliamentary Commissioner for the Environment, 2012; Todd Energy, 2012). Subsequently, energy companies have been granted exploratory permits for the east coast of North and South Island where shale resources are suspected; these areas are reportedly “poised on the brink of what could be a larger and rapid expansion of oil and gas production” (Parliamentary Commissioner for the Environment, 2012, p. 29). Even following a 2011 tightening of environmental and health and safety regulation surrounding fracking operations in New Zealand, controversy has hardly eased (Parliamentary Commissioner for the Environment, 2014).

A multi‐method approach was adopted, using interviews, diagrammatic analysis, and newsprint media analysis. To a certain extent, this three‐pronged approach minimized the impact of limitations associated with any single method in isolation. For example, interviews were useful because they captured the views of, and framings considered by, elite stakeholders in‐depth. For certain stakeholders, this may allow personal views to emerge that may have been left out of published documents. In a more extreme case, interviewing provides the opportunity to collect opinions from individuals who lack access to newsprint or diagrammatic communication platforms. It further allowed multiple views about fracking to be investigated, namely, from stakeholders, the media, and the policy community. The necessity of the multi‐method approach employed by this study lies in the reality that applying just one approach provides only a partial account of discussions relating to fracking operations in New Zealand. Considering this, all three approaches and the results they generate inform the discussion in a synergistic manner, rather than certain methods applying to specific scales of analysis.

### 3.1. Primary Data

Primary data were collected using semi‐structured interviews. This allowed a focus on elite stakeholders, which have been identified as important in framing and shaping controversies (Plutzer, Maney, & O'Connor, 1998; Sica, 2015). Consequently, the interviews indicate the concerns associated with fracking from the perspective of elite stakeholders, as opposed to “direct” assessment of public attitudes that questionnaire surveys have sought to achieve elsewhere (Boudet et al., 2014; Whitmarsh et al., 2015; Williams, Macnaghten, Davies, & Curtis, 2015).

A purposive sampling technique was employed, allowing stakeholders to be targeted. Interviewees were selected from websites of institutions involved in conducting, regulating, researching, or opposing fracking operations in New Zealand. Additional interviewees were identified through snowballing. The continuous referral to certain individuals indicates comprehensive coverage of stakeholders across the spectrum of interest.

Twenty‐five semi‐structured interviews were undertaken with 28 participants between 13/08/15 and 16/09/15. Interviews ranged from 25 to 145 minutes and were conducted face‐to‐face (19/25), via telephone (3/25), and video link (3/25). Interviewees were classified into a range of stakeholder types and regional geographies (supplementary material). Transcribed interviews were analyzed using a thematic hierarchical coding technique (Strauss, 1987) with subsequent quantitative analysis (supplementary material). Each interview was read multiple times to ensure codes emerged from the data rather than imposing predetermined codes (Cope, 2010). That said, given the transcripts were self‐generated artifacts, the themes on which the interview questions were based did feature during coding (Cope, 2010). Codes were built into themes by reading across interviews. This was achieved by creating documents into which similar codes from each interview were collated and reanalyzed (Bryman, 2012). Limited sample size meant the results of these quantitative analyses are not statistically significant. Nevertheless, they do indicate the issues considered important by those interviewed.

### 3.2. Secondary Data

#### 3.2.1. Diagrammatic Representations of Fracking

This study compares three diagrams identified from a preliminary survey of policy documents and internet resources regarding fracking in New Zealand. The selected diagrams (Fig. 3) originate from activist, industry, and expert advisory groups, respectively. Though three diagrams cannot represent the full variety of stakeholder types, critical attention towards those presented here provides an insight to the varied ways in which diagrams may be employed for risk communication. The approach to image analysis focused on the site of image production and on the image itself, with reference to Rose's (2013) three modalities: technological (selecting objects designed to be consumed visually), compositional (considering content, color, and presentation of the image), and social (acknowledging economic, social, and political relations, institutions and practices that surround an image, and through which it is seen and used). This formalized approach helped to minimize the influence of positionality in the interpretation of the diagrams (Rose, 1997).

#### 3.2.2. Newsprint Media Analysis

A Factiva search was performed on New Zealand's three most‐read print newspapers for articles including the term “fracking” and published between 01/09/10 and 01/10/15. “Fracking” was chosen as opposed to “hydraulic fracturing” because it is used specifically to refer to fracking in the context of oil and gas. The Factiva search returned 454 print newspaper articles with mention of the term “fracking,” which were subjected to an automated content analysis using Atlas.ti (supplementary material). While it is not possible to distinguish whether articles refer to fracking in New Zealand or in other national contexts, this media analysis provides an important insight to the temporal evolution of the debates.

## 4. RESULTS AND DISCUSSION

### 4.1. Fracking Controversy in New Zealand

**Figure 1:**
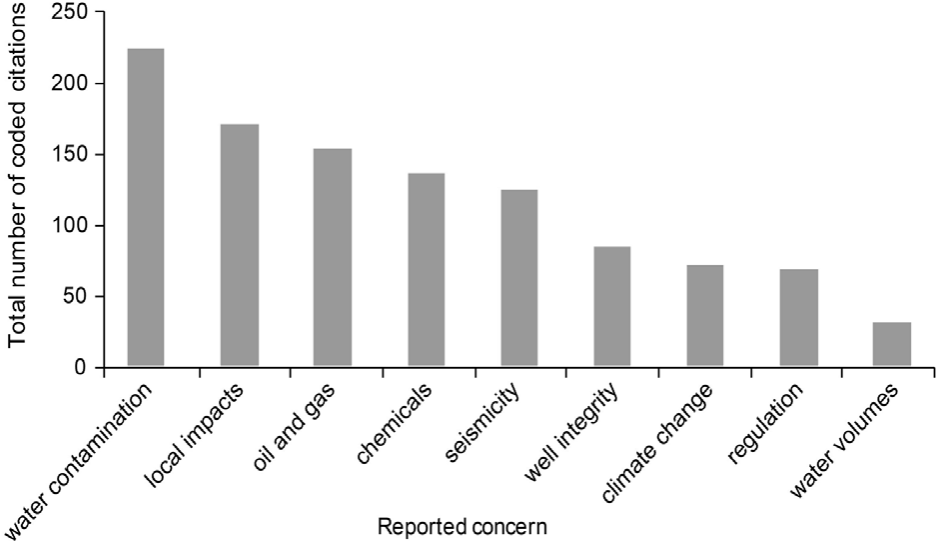
Total number of coded citations of concerns from interviews associated with fracking in New Zealand.

**Figure 2:**
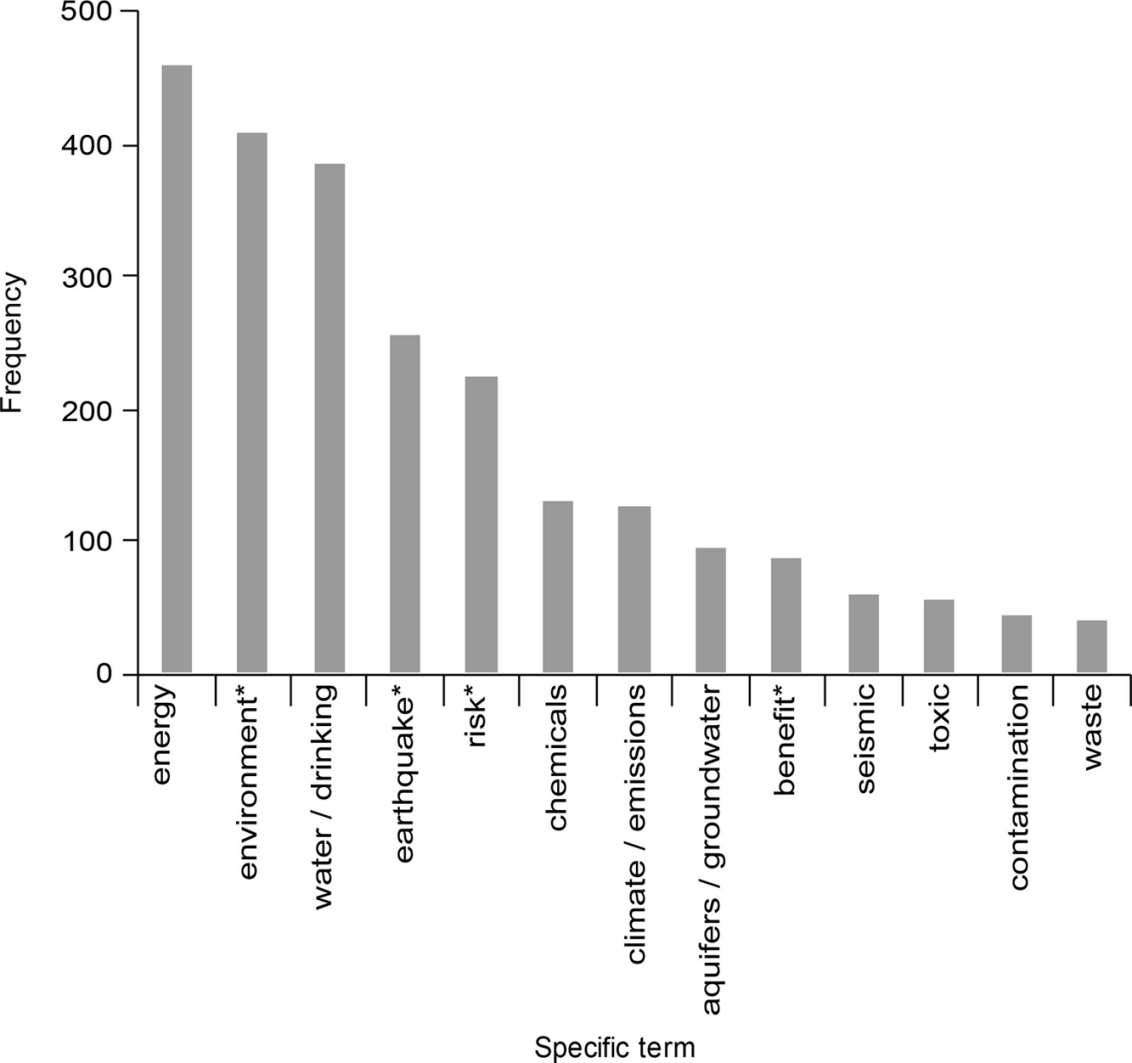
Total number of citations of specific terms in newsprint articles including the term “fracking” in New Zealand's three most‐read print papers. “*” Indicates truncated words, e.g. “earthquake*” includes counts of both “earthquake” and “earthquakes.”

The controversy surrounding fracking in New Zealand represents a contest between different stakeholders seeking to impose their own interpretation of the important issues. The majority of risks identified during interviews and newsprint media analysis are social and environmental impacts of fracking (Figs. 1 and 2). These risks were frequently positioned against economic benefits, setting up a familiar dichotomy observed elsewhere with regard to fracking (Sica, 2015; Whitmarsh et al., 2015). Scientific reports, media statements, and political remits (local councils are not allowed to consider climate change during resource consenting) simultaneously define the dominant issue as environmental risk while assuring stakeholders that this risk is manageable, or at least outweighed by economic benefits. The advantages of this derive from the exclusion from regulatory discussions, broader issues that are more intractable and less easily addressed through familiar environmental risk assessment approaches.

**Figure 3:**
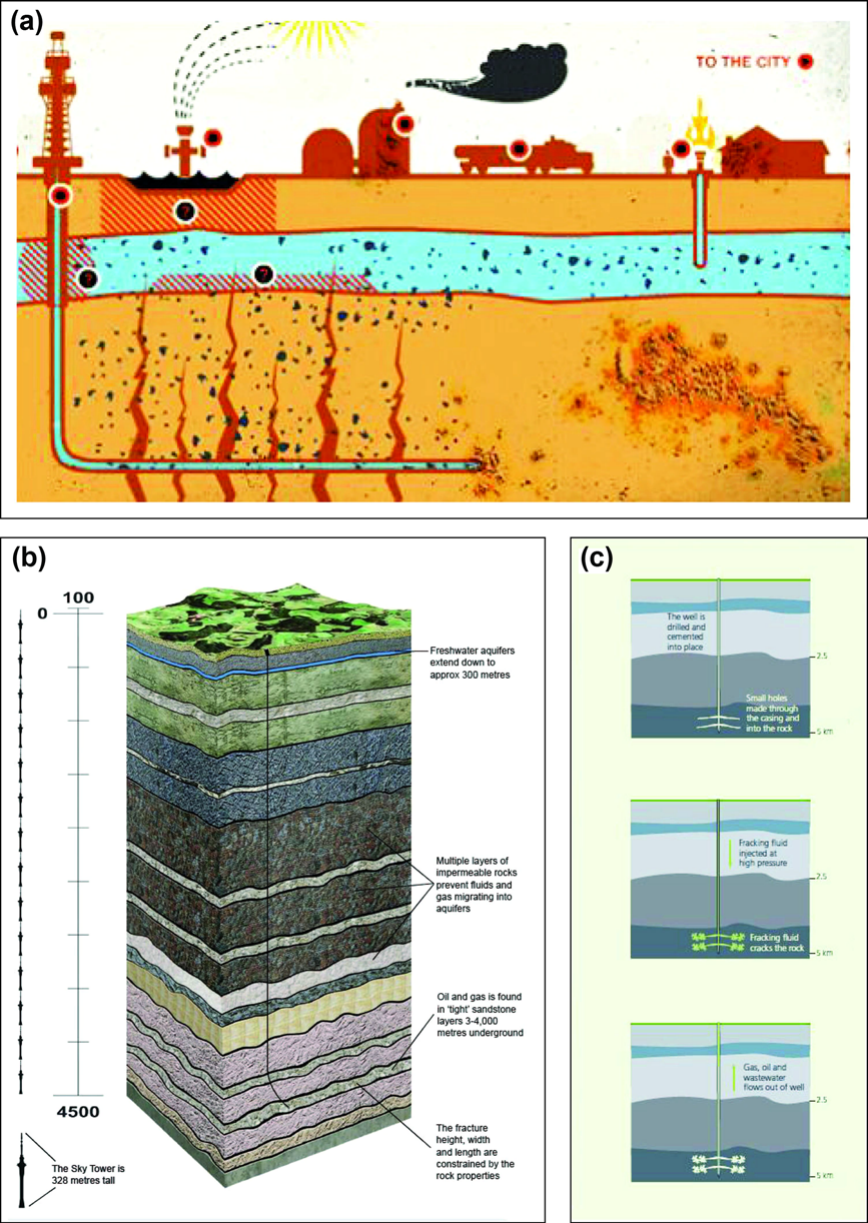
Diagrammatic representations of fracking in New Zealand publications. (a) “What is Fracking?” on Lock the Gate Aotearoa website; (b) Fracking—An introduction to operations in Taranaki, Todd Energy; (c) The main stages of cracking rocks so oil and gas can flow (PCE, 2012).

The contest between different stakeholders is also visually evident as demonstrated through diagrammatic analysis (Fig. 3). This is an important aspect of risk perception given that opinion formation is also guided by “top‐of‐mind” associations regarding the affective qualities of risk information (Finucane, Alhakami, Slovic, & Johnson, 2000). On first inspection, the Parliamentary Commissioner for the Environment (PCE) diagram (Fig. 3c) appears neutral; yet a choice has been made in the use of muted colors and in presenting deep fracturing. An alternative technique is deployed in the Todd Energy diagram (Fig. 3b) where a concerted effort has been made to emphasize the distance between the fracturing and the aquifer layers using the Auckland Sky Tower to communicate relative distances. Both diagrams indicate the small dimensions of fractures in relation to the overall operation. In this way, Figs. 3b and 3c intend to depoliticize environmental risks that have gained significant attention in the fracking debate. By contrast, the image from the Lock the Gate Aotearoa website (Fig. 3a) emphasizes the threats posed to groundwater showing fractures extending into the aquifer layer. There is also a visual representation of flammable water sources—an iconic anti‐fracking image. These images reinforce associations between specific risks and fracking, contributing to the way fracking is constructed in the cognitive spaces of stakeholders (Finucane et al., 2000; Rose, 2013; Slovic, 1999). It is against this backdrop of discursive and visual conflict that we will explore the multi‐scalar influences on risk perception surrounding fracking in New Zealand.

### 4.2. International

High‐profile events have the potential to reinforce or disrupt the risk perceptions of fracking outlined thus far. They draw attention to certain aspects of the debate and obscure others through amplification and attenuation effects. This study identifies two international‐scale events that exerted a notable impact on the evolution of debates in New Zealand, namely, the *Gasland* documentary and the Macondo oil spill.

On 15 September 2010, Josh Fox released the documentary *Gasland* about fracking in the United States The documentary was identified as influential to the New Zealand debate in >95% of the 25 interviews. Respondents frequently referred to one scene in which someone is shown setting their kitchen tap on fire, providing a convincing illustration of the social amplification of risk framework (e.g., Fig. 3a). Continuing with the SARF terminology, *Gasland* can be considered a “risk event” with a notable influence on the “risk signal” (Kasperson et al., 2003) of fracking. This has multi‐scale impacts, with subsequent ripple effects for third parties, e.g., regional councils who must deal with concerns conceived though comparisons between the United States and New Zealand. Furthermore, *Gasland*'s focus on water contamination, as noted elsewhere (Cooper & Nisbet, 2016; Vasi et al., 2015), appears to resonate with regionally‐specific water‐related concerns. This was evident in both the coded interviews and media analysis where water contamination dominated citations, accounting for 21% and 16% of total citations, respectively (Figs. 1 and 2).

The Macondo oil spill further demonstrates the potential for international events to shape risk perception at finer spatial scales. Cited in 36% of interviews as shaping national debates, the Macondo spill, much like disasters associated with the nuclear industry, exemplifies the catastrophic potential of oil and gas extraction (Pidgeon & Demski, 2012; Visschers & Siegrist, 2013).

there have [been] some very high, tragic catastrophes, whether it's North Sea, Gulf of Mexico, with significant loss of life and pollution when human error occurs. And it causes community concern, because when it goes wrong, it can go wrong in a catastrophic sense. (Expert Advisory Body)

The attention gained by fracking might then be interpreted as the result of a two‐way relationship where association with the oil and gas industry intensifies fears about the impacts of fracking, with fracking subsequently providing a focal point for fears over oil and gas. Although “violations” have been documented in the conduct of fracking (Moniz et al., 2011), no globally catastrophic events with direct links to fracking operations have occurred. Consequently, this study's finding that in the majority of instances, catastrophic events were associated with fracking regardless, is arguably indicative of a shared reputation across the oil and gas industry (Small et al., 2014).

**Figure 4:**
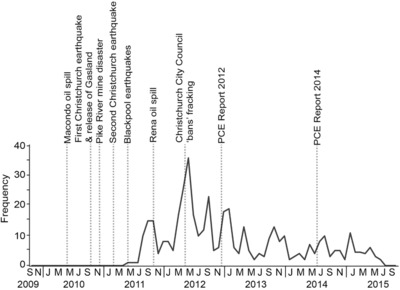
Frequency of newsprint articles including the term “fracking” in New Zealand's three most‐read print papers, October 2009–October 2015.

Tangible links between fracking and oil and gas markets can also calm debates, as demonstrated in Fig. 4, with newsprint media mentions of “fracking” tailing off towards the end of 2015. One explanation is the plummeting global oil price and associated decline in the economic feasibility of fracking. This short‐term lapse of attention could, perhaps, provide an opportunity to incorporate some of the less tangible concerns that stakeholders in the debate have expressed.

### 4.3. National

Four national influences emerging from data analysis are elaborated upon. Comparisons with the geothermal energy and agricultural industries are used to explain the “lightning rod” metaphor, then the Christchurch earthquake and the PCE 2012 Report publication are posited as important national events that shaped risk perceptions.

The “lightning rod” metaphor refers to use of fracking by certain stakeholders to draw attention to oil and gas operations as a whole in New Zealand, as well as underlying concerns surrounding climate change. These broader issues accounted for 15% and 7%, respectively, of coded citations from interviews (Fig. 1). The importance of the wider context in which a given risk is situated has been noted elsewhere in relation to emerging technologies. For example, the risks of radiation are perceived as greater in the context of nuclear waste than in medical practices (Flynn, 2003). This study suggests that a contextual influence on risk perception is evident when fracking in the oil and gas industry is compared to similar techniques in geothermal power generation. Several respondents noted that geothermal fracturing in New Zealand has not attracted the widespread attention associated with fracking. This is likely attributable, in part, to differences between the techniques employed in each industry, as geothermal operations typically occur in areas of greater natural seismicity, far from populations, and on a smaller scale compared to oil and gas operations. This study suggests an additional reason geothermal fracturing has gained less attention than oil and gas fracking is stigmatism of the latter industry.

Continuing the discussion of national issues that underwrite the fracking debate, some respondents looked beyond the oil and gas industry, positing climate change as the underlying concern. A second industry comparison, this time with agriculture, brings into question the association of climate change with fracking in New Zealand. Agriculture appears to enjoy comparatively little stigma, given that it accounted for the greatest percentage of occupational fatalities (43%) in New Zealand in 2015 (Worksafe, 2015) and contributed the greatest percentage of any industry to greenhouse gas emissions (48%) in 2013 (New Zealand Ministry for the Environment, 2015). One respondent sought to provide an explanation for this:

Interviewer: Does the farming sector get much flack for climate change issues?

…there's a general perception that farming was a good… years ago perhaps when I was a child… farming was a good thing to do, it's kind of archetypical New Zealand, Kiwis like the farming sector and all that. (Local Government)

While associations between agriculture and methane emissions are well documented, the link between fracking and climate change is contested (Feng, Davis, Sun, & Hubacek, 2016; Stephenson, Doukas, & Shaw, 2012). It is possible to identify two distinct narratives. The first asserts that fracking perpetuates dependence on fossil fuels by enabling the exploitation of resources unattainable with conventional drilling techniques. The second is widely referred to as the “transition fuel argument” and purports the view that natural gas, accessed through fracking, provides a stepping stone between dirtier hydrocarbons and renewables that are not yet commercially viable (Stephenson et al., 2012). This latter narrative might be described as a legitimizing discourse (Hajer, 1997), employed to obscure the climate risk associated with fracking. In the context of New Zealand, both narratives are, arguably, of limited relevance given that gas contributes just over 20% to the current energy mix (Gas Industry Company, 2014). Both narratives are employed regardless, perhaps reflecting an awareness of the momentum gained by the global climate change movement and the potential benefit of aligning fracking with it.

Moving on from industry comparison to national risk events, the greatest media coverage surrounding fracking corresponds with the Christchurch City Council's decision to place a moratorium on fracking operations on April 12, 2012, following earthquakes in September 2010 and February 2011 (Fig. 4). The council's decision is intriguing for the reasons articulated by one respondent:

Yep, the Christchurch City Council, having been through the earthquakes, banned fracking, yeah it was actually a huge joke because a) nobody was actually talking about doing any fracking anywhere near Christchurch or anywhere in the Christchurch area anyway b) the City Council has no power to ban fracking… (Local Government).

This paints the council's decision as a political move, though not unfounded given the widespread trauma and destruction caused by the earthquakes. A comparison can be drawn between the interaction of the Christchurch earthquake with fracking and the Sendai earthquake with nuclear power. The Fukushima disaster exemplifies a natural disaster colliding with a techno‐environmental risk in a tangible sense, through tsunami‐induced flooding; whereas the Christchurch earthquake collided with fracking only in the intangible discursive space and no fracking wells were compromised. Yet because the earthquakes that hit Christchurch entered a context where fracking practices had been linked to induced seismicity in the United States (Moniz et al., 2011), risk amplification occurred, with ripple effects reaching the Taranaki region.

Germany was doing it, France was doing it, various [parts] of the States were doing it, these sort of moratoria for a period. And Christchurch City Council has done it, why hasn't Taranaki Regional Council banned it —that was the logic. (Local Government)

Intriguingly, despite national risk amplification, the Christchurch earthquake had little, if any, influence on the international fracking debates, unlike Fukushima, which triggered international policy responses, such as in Germany.

In contrast to the above example, national events are not exclusively risk amplifying. This is exemplified by the November 2012 PCE Report publication (Parliamentary Commissioner for the Environment, 2012). The report, which investigated exclusively the environmental impacts of fracking, concluded that a moratorium was not justified given the current state of scientific evidence. This attenuated concern because of the credibility associated with the PCE itself (Hilgartner, 2000). The Commissioner is considered well trusted by virtually all respondents, primarily deriving from an independent status.

I thought that was a very good piece of work and it's befitting of one who sits independent of government and just tries to provide the best advice without political interference. (Expert)

There was aspects of the PCE that I think gave some of our community stakeholders some piece of mind, that an independent review had found, had the same conclusion that we do as a company. (Industry)

Even though risk assessments by the Taranaki Regional Council, Todd Energy, and GNS Science informed the PCE report, as stand‐alone sources, they lacked the credibility required to achieve widespread influence. One contributory factor is the New Zealand regulatory context, which states that the regional government is responsible for regulation of environmental laws *and* facilitating economic activity, presenting a potential conflict of interests. Furthermore, given that industry was expected to fulfill a self‐regulating role, evidence produced concerning the environmental impacts of fracking activities engendered notable skepticism. Where trust in the source of information is lacking, a focus on communicating scientific evidence from environmental risk assessment is often ineffective, given that results may be dismissed out of hand because of prior beliefs about the credibility of organizations involved (Eiser, Stafford, Henneberry, & Catney, 2009; Petts, 2008).

### 4.4. Local and Regional

Local and regional influences are jointly considered, with “local” referring to locations within Taranaki where fracking is taking place and “regional” referring primarily to Hawkes Bay, where fracking operations are prospective.

Virtually all approaches to risk assessments focus on the environmental risks and are locally specific to Taranaki (Parliamentary Commissioner for the Environment, 2012, 2014; Sherburn & Quinn, 2012; Taranaki Regional Council, 2012; Todd Energy, 2012). Discussions with government regulators and policymakers revealed a persistent belief in the need to educate the public by “filling the information void” with evidence concerning the environmental impacts of fracking operations. These discussions typically converged around the term “well integrity,” which was frequently deployed to demonstrate the manageable nature of the identified risks.

If you get your well integrity and hydrogeological integrity right…piece of cake. (Local Government)

This represents a “closing down” (Stirling, 2008) of policy options—the question becomes not whether fracking is desirable, but how best to manage its impacts. Environmental risk assessments remain important and will play a critical role in assessing the tangible risks that fracking poses. Yet the prospect of oil and gas industry expansion to Hawkes Bay, facilitated by fracking, and justified by environmental risk assessments predicated in the Taranaki context has triggered widespread and enduring concern. This suggests an inability of nonlocal risk assessment, and subsequent risk communication focusing on the concept of “well integrity,” to alleviate stakeholder concerns.

**Figure 5:**
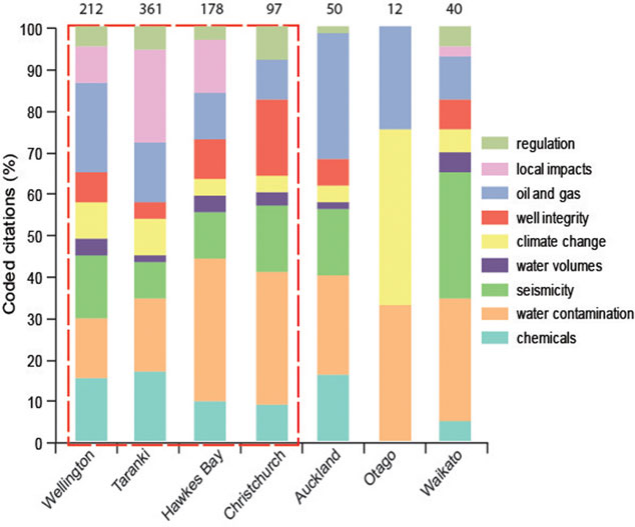
Coded citations of concerns associated with fracking by regional geography. Figures atop columns indicate total number of coded citations. Red box indicates regions with >3 interviewees.

In material terms, Hawkes Bay draws the majority of its wealth from high‐value horticultural activities. This places huge importance on the aquifer and helps to explain why water contamination accounted for 35% of citations in Hawkes Bay, the most of any region (Fig. 5). Additionally, in contrast to Taranaki, where fracking has been implemented since 1989, activity in Hawkes Bay is only prospective. In Hawkes Bay, neither communities nor local councils have experience of the oil and gas industry, while in Taranaki:

We've been dealing with oil since 1860‐something in Taranaki, we know it, it is not an ogre, it is not something demonic, it's something you deal with and regulate and the industry actually performs incredibly well in terms of its level of compliance. (Local Government)

Taranaki's history of oil and gas production is perceived as a defining characteristic of this area's identity to this day. Consequently, a significant proportion of the population is likely to know someone, or themselves be, employed in this industry. This is pertinent given in the United States it has been found that support for fracking is more prevalent in locations where a significant proportion of the population is employed in the extractive industry (Boudet et al., 2016).

While geological differences between regions can be addressed using existing environmental risk management techniques, social and cultural differences cannot (Wynne, 1992). The perceived novelty of a given technology is an important psychometric characteristic (Fischhoff et al., 1978; Slovic, 1987). There is ongoing debate as to whether fracking can be considered a novel technology and this was mirrored in the interviews conducted. The consensus appeared to be that novelty was acquired in certain contexts, including the prospect of expansion to Hawkes Bay, and to some extent Canterbury, where there is no historic precedent of oil and gas.

what the fracking does is it means we can now drill wells in Gisborne or Canterbury or wherever we couldn't before. So people who have never had to deal with an oil company before or a council who have never had to understand the risks of an oil well. (Expert Advisory Body)

In addition to being a regionally novel technology, fracking activities conflict with the well‐established place‐based identity of Hawkes Bay. This identity revolves around its role as the “fruit bowl” of New Zealand. The “clean green” image of New Zealand appears amplified here given the reputation of the region as the primary producer of fruit and vegetables for New Zealand.

Arguably the best quality land in terms of the soil that we've got in the country, that's why it's the fruit bowl and it's got the best quality water so it draws water. The whole raison d'etre for Hawkes Bay really is that fruit bowl and this is just madness to put that at risk, frankly its madness. (Activist)

In this instance, opposition to fracking is predicated not only on concerns about environmental risks, but also on the possibility that the very identity of the region will change because of these activities. The inability of the current approach to build legitimacy or trust among a concerned citizenry becomes a central impediment to the acceptance of emerging technologies, with the potential to seriously undermine relationships between stakeholders.

### 4.5. Applying Risk Perception to Understand SLO Outcomes

The discussion so far has established that the controversy surrounding fracking in New Zealand is the result of numerous multi‐scalar influences on risk perception, which have led to conflict between stakeholders. Yet, it is untrue to say that there have been no efforts to incorporate a wider array of voices into risk assessment and management. The foremost attempt to achieve more democratic stakeholder engagement is manifest in the pursuit of a “social license to operate” in Taranaki by fracking operators. Interviews revealed that while endorsement was not universal among respondents, there was reasonable consensus on the type of engagement required, including: building trust and credibility; responsible business practice; being a good neighbor; and fostering active engagement. These characteristics are in broad agreement with literature on social license (Prno & Slocombe, 2012).

In practice, there is a bias towards addressing SLO at the local scale in isolation of multi‐scale variables, that have been identified as importance influences on SLO outcomes in the mining industry literature (Parsons et al., 2014; Prno & Slocombe, 2014). This is exemplified in Taranaki where local impacts accounted for 23% of citations in the region (Fig. 5). Having identified that social license is unfulfilled, operators in Taranaki displayed concerted efforts to address the issues raised. For example, in response to complaints over trucking, water for use in fracturing operations was pumped onto the site. The operator's wider intention is articulated by the following statement:

what we wanted to do is be just the same as a farmer or resident and be available 24 hour a day, there was a big piece around a 0800 number so people can ring the company, 24 hours a day whenever they are experiencing anything related to our operation. (Industry)

This kind of engagement is necessary, but also revealing. Applied in its present form, the SLO seeks to engage citizens only when they are perceived to be directly impacted by the fracking operations. As a result, those experiencing tangible local impacts of fracking in Taranaki have been engaged by operators since straightforward solutions can be identified, e.g., limiting trucking and flaring to certain hours. By contrast, those in Hawkes Bay have not been actively engaged. This reflects the prospective status of operations there and lacking recognition of less tangible psychometric and place‐based factors.

The preceding analysis of national‐scale factors suggests a substantive influence on risk perception. Industry comparisons between fracking, geothermal energy, and agriculture draw attention to national‐scale policy decisions regarding energy mix climate change that are already been enshrined in policy. For obvious economic and political reasons, it is often not feasible to reopen these broader debates, yet simply obscuring them by situating engagement at the local level or dismissing them as out of remit is unlikely to foster productive relationships between stakeholders. Extending this argument to international focusing events, the need to engage with “risk events” such as *Gasland* was recognized by some stakeholders, but not others.

Gas Lands is about America so we can't say well actually the truth in America was, you know, that is not our expertise, we know about Taranaki, we're experts in Taranaki. (Regulator)

Given that stakeholders involved in the fracking industry cannot control these events, communicating the relevance of international influences to national, regional, and local concerns should be a priority. When the implementation of SLO does not live up to the associated rhetoric, it fosters highly skeptical attitudes:

I think it's [social license] enabling particular types of behaviours and disabling others. It pays lip service to some things. (Expert Advisory Body)

This critical judgment suggests the social license to operate could be invoked by operators to claim legitimacy of fracturing operations in the absence of truly inclusionary processes. This has serious implications regarding the democratic basis of decisions on fracking in New Zealand.

## 5. CONCLUSIONS

This study offers two contributions to the understanding of risk perceptions surrounding fracking and the implications for risk management outcomes. First, it considers the determinants of fracking risk perception using a multi‐scalar approach with attention to the intra‐scalar interactions and their shaping of risk perception. Building on previous studies that utilize constructivist theory to explain fracking controversy (Boudet et al., 2014; Clarke et al., 2015), it is argued that the multi‐scalar analysis undertaken here provides a fuller appreciation of the range of factors, and to some extent their relative importance in New Zealand. The intra‐scalar interaction between these factors is clearly demonstrated by the *Gasland* documentary, an international “risk event” whose focus on water contamination resonates with regional concerns regarding aquifer contamination in Hawkes Bay, and that triggered the need for local engagement from fracking operators and regulators in Taranaki. *Gasland*'s influence on debates at finer scale reflects a failure of stakeholders to acknowledge the relevance of international influencing factors and engage proactively with concerns emanating from them. National industry comparisons reveal that fracking acts as a lightning rod, drawing attention to national policy surrounding the oil and gas industry and climate change. Favorable reception of publications from the PCE, derived from its perceived independence and credibility, perhaps suggests that this is the most appropriate institution for engaging with these national issues.

Second, this study applies a multi‐scalar understanding of risk perception to compose a critique of the SLO concept as applied to fracking. In both mining and fracking, there is evidence that certain stakeholders consider SLO to apply locally without consideration of alternative scales (Parsons et al., 2014). This is demonstrated by the inability of SLO to address less tangible concerns triggered by prospective fracking in Hawkes Bay. Locally specific environmental risk assessments appear to lack the perceived generality that is required if they are to be successful in attenuating regional concerns. The conceptual framework developed by Prno and Slocombe (2014) provides a basis for comprehensive multi‐scalar analysis of SLO outcomes, yet only mentions the relevance of “perception” in passing. The results of this study suggest that communities reserve their “social license” if they perceive the risks associated with an activity to outweigh the associated benefits. Incorporating the multi‐scalar influences on risk perception is, therefore, an essential addition to this framework.

The emerging nature of hydraulic fracturing and attempts to manage it give rise to numerous avenues for future research. This study has found that regional and national‐scale influences are especially important in determining risk perceptions in the New Zealand context, yet it is unknown whether this is typical elsewhere in the world. Clarification here could reveal whether engagement at these levels ought to be prioritized before intervening locally. Moving on to international influences, this article agrees with previous work into the global nature of media events such as *Gasland* (Mazur, 2014; Vasi et al., 2015), but it is unknown whether this is an Anglophone phenomena or a truly international influence. Finally, it is interesting to note that the Christchurch earthquake did not have an international risk amplifying effect despite the high‐profile nature of the disaster. It would be interesting to investigate the reasons for this “failed amplification.”

Notwithstanding the contribution of this study, it is necessary to recognize some limitations. Although the constructivist lens provides a convincing explanation for controversy, in practice, it is difficult to incorporate an appreciation of these factors in a consistent manner. More research (ideally in collaboration with practitioners) is required to provide implementable guidance that encapsulates the insights offered here and in frameworks such as that of Prno and Slocombe (2014). Such insights must be considered alongside environmental risk assessments and economic appraisals in recognition that these approaches also make valuable contributions to the understanding of risk.

## Supporting information

Supplementary Material

## References

[risa13167-bib-0001] Beck, U. (1992). Risk society: Towards a new modernity. California: SAGE Publications Ltd.

[risa13167-bib-0002] Boudet, H. S. , Bugden, D. , Zanocco, C. , & Maibach, E. (2016). The effect of industry activities on public support for “fracking.” Environmental Politics, 25(4), 593–612.

[risa13167-bib-0003] Boudet, H. , Clarke, C. , Bugden, D. , Maibach, E. , Roser‐Renouf, C. , & Leiserowitz, A. (2014). “Fracking” controversy and communication: Using national survey data to understand public perceptions of hydraulic fracturing. Energy Policy, 65, 57–67.

[risa13167-bib-0004] Boutilier, R. G. (2014). Frequently asked questions about the social licence to operate. Impact Assessment and Project Appraisal, 32(4), 263–272.

[risa13167-bib-0005] Boutilier, R. G. , & Thomson, I. (2011). Modelling and measuring the social license to operate: Fruits of a dialogue between theory and practice. Social Licence to Operate. Available at https://socialicense.com/index.html

[risa13167-bib-0006] Bryman, A. (2012). Social research methods (4th ed). Oxford: Oxford University Press.

[risa13167-bib-0007] Clarke, C. E. , Hart, P. S. , Schuldt, J. P. , Evensen, D. T. N. , Boudet, H. S. , Jacquet, J. B. , & Stedman, R. C. (2015). Public opinion on energy development: The interplay of issue framing, top‐of‐mind associations, and political ideology. Energy Policy, 81, 131–140.

[risa13167-bib-0008] Cooper, K. E. , & Nisbet, E. C. (2016). Green narratives: How affective responses to media messages influence risk perceptions and policy preferences about environmental hazards. Science Communication, 38(5), 626–654.

[risa13167-bib-0009] Cope, M. (2010). Coding transcripts and diaries In Clifford N., French S., & Valentine G. (Eds.), Key methods in geography. California: SAGE Publications Ltd.

[risa13167-bib-0010] Cotton, M. (2013). NIMBY or not? Integrating social factors into shale gas community engagements. Natural Gas & Electricity, 29(9), 8–12.

[risa13167-bib-0011] Dake, K. (1992). Myths of nature: Culture and the social construction of risk. Journal of Social Issues, 48(4), 21–37.

[risa13167-bib-0012] Davis, C. , & Fisk, J. M. (2014). Energy abundance or environmental worries? Analyzing public support for fracking in the United States. Review of Policy Research, 31(1), 1–16.

[risa13167-bib-0013] Dear, M. (1992). Understanding and overcoming the NIMBY syndrome. Journal of the American Planning Association, 58(3), 288–300.

[risa13167-bib-0014] Devine‐Wright, P. (2009). Rethinking NIMBYism: The role of place attachment and place identity in explaining place‐protective action. Journal of Community and Applied Social Psychology, 19(6), 426–441.

[risa13167-bib-0015] Douglas, M. (1992). Risk and blame: Essays in cultural theory. London: Routledge.

[risa13167-bib-0016] Douglas, M. (1966). Purity and danger—An analysis of the concepts of pollution and taboo. purity and danger: An analysis of the concepts of pollution and taboo. London: Routledge.

[risa13167-bib-0017] Eden, S. (1998). Environmental issues: Knowledge, uncertainty and the environment. Progress in Human Geography, 22(3), 425–432.

[risa13167-bib-0018] Eiser, J. R. , Stafford, T. , Henneberry, J. , & Catney, P. (2009). “Trust me, I'm a scientist (not a developer)”: Perceived expertise and motives as predictors of trust in assessment of risk from contaminated land. Risk Analysis, 29(2), 288–297. 18826417 10.1111/j.1539-6924.2008.01131.x

[risa13167-bib-0019] Feng, K. , Davis, S. J. , Sun, L. , & Hubacek, K. (2016). Drivers of the US CO_2_ emissions 1997–2013. Nature Communications, 6(7714), 8. 10.1038/ncomms8714 PMC4518269 26197104

[risa13167-bib-0020] Finucane, M. L. , Alhakami, A. , Slovic, P. , & Johnson, S. M. (2000). The affect heuristic in judgments of risks and benefits. Journal of Behavioral Decision Making, 13(1), 1–17.

[risa13167-bib-0021] Fischhoff, B. , Slovic, P. , Lichtenstein, S. , Read, S. , & Combs, B. (1978). How safe is safe enough? A psychometric study of attitudes towards technological risks and benefits. Policy Sciences, 9(2), 127–152.

[risa13167-bib-0022] Flynn, J. (2003). Nuclear stigma In Pidgeon N. F., Kasperson R. E., & Slovic P. (Eds.), Social amplification of risk. Cambridge: Cambridge University Press.

[risa13167-bib-0023] Gas Industry Company . (2014). *The New Zealand gas story* .

[risa13167-bib-0024] Graham, J. D. , Rupp, J. A. , & Schenk, O. (2015). Unconventional gas development in the USA: Exploring the risk perception issues. Risk Analysis, 35(10), 1770–1788. 26460730 10.1111/risa.12512

[risa13167-bib-0025] Gunningham, N. , Kagan, R. A. , & Thornton, D. (2004). Social license and environmental protection: Why businesses go beyond compliance. Law & Social Inquiry, 29(2), 307–341.

[risa13167-bib-0026] Hajer, M. A. (1997). The politics of environmental discourse: Ecological modernization and the policy process. Oxford: Oxford University Press.

[risa13167-bib-0027] Halaweh, M. (2013). Emerging technology. What is it? Journal of Technology Management and Innovation, 8(3), 108–115.

[risa13167-bib-0028] Harvey, B. , & Bice, S. (2014). Social impact assessment, social development programmes and social licence to operate: Tensions and contradictions in intent and practice in the extractive sector. Impact Assessment and Project Appraisal, 32(4), 327–335.

[risa13167-bib-0029] Hilgartner, S. (2000). Science of stage: Expert advice as public drama. Stanford, CA: Stanford University Press.

[risa13167-bib-0030] House, E. J. (2013). Fractured fairytales: The failed social license for unconventional oil and gas development. Wyoming Law Review, 13(1), 5–67.

[risa13167-bib-0031] Hu, D. , & Xu, S. (2013). Opportunity, challenges and policy choices for China on the development of shale gas. Energy Policy, 60, 21–26.

[risa13167-bib-0032] International Energy Agency . (2011). World energy outlook 2011 special report—Are we entering a golden age of gas? Paris, France: IEA.

[risa13167-bib-0033] Jasanoff, S. (1991). The fifth branch: Science advisers as policymakers. Cambridge: Harvard University Press.

[risa13167-bib-0034] Kahan, D. M. , Braman, D. , Slovic, P. , Gastil, J. , & Cohen, G. (2009). Cultural cognition of the risks and benefits of nanotechnology. Nature Nanotech, 4, 87–90. 10.1038/nnano.2008.341 19197308

[risa13167-bib-0035] Kasperson, J. X. , Kasperson, R. E. , Pidgeon, N. , & Slovic, P. (2003). The social amplification of risk: Assessing fifteen years of research and theory In Pidgeon N. F., R. E. Kasperson, & Slovic P. (Eds.), The social amplification of risk. Cambridge: Cambridge University Press.

[risa13167-bib-0036] Kasperson, R. E. , Renn, O. , Slovic, P. , Brown, H. S. , Emel, J. , Goble, R. , … Ratick, S. (1988). The social amplification of risk: A conceptual framework. Risk Analysis, 8(2), 177–187.

[risa13167-bib-0037] Larock, S. , & Baxter, J. (2013). Local facility hazard risk controversy and non‐local hazard risk perception. Journal of Risk Research, 16(6), 713–732.

[risa13167-bib-0038] Mazur, A. (2014). How did the fracking controversy emerge in the period 2010–2012? Public Understanding of Science, 25(2), 207–222. 25106618 10.1177/0963662514545311

[risa13167-bib-0039] Moffat, K. , & Zhang, A. (2014). The paths to social licence to operate: An integrative model explaining community acceptance of mining. Resources Policy, 39(1), 61–70.

[risa13167-bib-0040] Moniz, E. J. , Jacoby, H. D. , & Meggs, A. J. M. (2011). The future of natural gas: An interdisciplinary MIT study. MIT Energy Initiative, 170, 1–308.

[risa13167-bib-0041] Montgomery, C. T. , & Smith, M. B. (2010). Hydraulic fracturing—History of an enduring technology. Journal of Petroleum Technology, 62(12), 26–41.

[risa13167-bib-0042] New Zealand Ministry for the Environment . (2015). *New Zealand's greenhouse gas inventory 1990–2013* .

[risa13167-bib-0043] Owen, J. R. , & Kemp, D. (2013). Social licence and mining: A critical perspective. Resources Policy, 38(1), 29–35.

[risa13167-bib-0044] Owens, S. (2000). “Engaging the public”: Information and deliberation in environmental policy. Environment and Planning – Part A, 32(7), 1141–1148.

[risa13167-bib-0045] Parliamentary Commissioner for the Environment . (2012). *Evaluating the environmental risks of fracking in New Zealand* .

[risa13167-bib-0046] Parliamentary Commissioner for the Environment . (2014). *Drilling for oil and gas in New Zealand: Environmental oversight and regulation* .

[risa13167-bib-0047] Parsons, R. , Lacey, J. , & Moffat, K. (2014). Maintaining legitimacy of a contested practice: How the minerals industry understands its “social licence to operate.” Resources Policy, 41(1), 83–90.

[risa13167-bib-0048] Perlaviciute, G. , & Steg, L. (2014). Contextual and psychological factors shaping evaluations and acceptability of energy alternatives: Integrated review and research agenda. Renewable and Sustainable Energy Reviews, 35, 361–381.

[risa13167-bib-0049] Petts, J. (2008). Public engagement to build trust: False hopes? Journal of Risk Research, 11(6), 821–835.

[risa13167-bib-0050] Pidgeon, N. F. , & Demski, C. (2012). From nuclear to renewable: Energy system transformation and public attitudes. Bulletin of the Atomic Scientists, 68(4), 41–51.

[risa13167-bib-0051] Plutzer, E. , Maney, A. , & O'Connor, R. E. (1998). Ideology and elites’ perceptions of new technology. American Journal of Political Science, 42(1), 190–209.

[risa13167-bib-0052] Prno, J. (2013). An analysis of factors leading to the establishment of a social licence to operate in the mining industry. Resources Policy, 38(4), 577–590.

[risa13167-bib-0053] Prno, J. , & Slocombe, D. S. (2014). A systems‐based conceptual framework for assessing the determinants of a social license to operate in the mining industry. Environmental Management, 53(3), 672–689. 24375075 10.1007/s00267-013-0221-7

[risa13167-bib-0054] Prno, J. , & Slocombe, S. (2012). Exploring the origins of “social license to operate” in the mining sector: Perspectives from governance and sustainability theories. Resources Policy, 37(3), 546–357.

[risa13167-bib-0055] Rose, G. (1997). Situating knowledges: Positionality, reflexivities and other tactics. Progress in Human Geography, 21(3), 305–320.

[risa13167-bib-0056] Rose, G. (2013). Visual methodologies: An introduction to the interpretation of visual materials (3rd ed.) California: SAGE Publications Ltd.

[risa13167-bib-0057] Rotolo, D. , Hicks, D. , & Martin, B. R. (2015). What is an emerging technology? Research Policy, 44(10), 1827–1843.

[risa13167-bib-0058] Ruckstuhl, K. , Thompson‐Fawcett, M. , & Rae, H. (2014). Māori and mining: Indigenous perspectives on reconceptualising and contextualising the social licence to operate. Impact Assessment and Project Appraisal, 32(4), 304–314.

[risa13167-bib-0059] Sandman, P. M. (1987). Risk communication: Facing public outrage. EPA Journal, 13, 21.

[risa13167-bib-0060] Severson, G. (2012). Public relations: Managing NIMBY issues before they manage you. Natural Gas and Electricity, 29(5), 18–22.

[risa13167-bib-0061] Sherburn, S. , & Quinn, R. (2012). An assessment of the effects of hydraulic fracturing on seismicity in the Taranaki region. Wellington, New Zealand.

[risa13167-bib-0062] Sica, C. E. (2015). Stacked scale frames: Building hegemony for fracking across scales. Area, 47(4), 443–450.

[risa13167-bib-0063] Slovic, P. (1987). Perception of risk. Science, 236(4799), 280–285. 3563507 10.1126/science.3563507

[risa13167-bib-0064] Slovic, P. (1999). Trust, emotion, sex, politics, and science: Surveying the risk‐assessment battlefield. Risk Analysis, 19(4), 689–701. 10765431 10.1023/a:1007041821623

[risa13167-bib-0065] Small, M. J. , Stern, P. C. , Bomberg, E. , Christopherson, S. M. , Goldstein, B. D. , Israel, A. L. , … Zielinska, B. (2014). Risks and risk governance in unconventional shale gas development. Environmental Science and Technology, 48(15), 8289–8297. 24983403 10.1021/es502111u

[risa13167-bib-0066] Starr, C. (1969). Social benefit versus technological risk. Science, 165(3899), 1232–1238. 5803536 10.1126/science.165.3899.1232

[risa13167-bib-0067] Stephenson, E. , Doukas, A. , & Shaw, K. (2012). Greenwashing gas: Might a “transition fuel” label legitimize carbon‐intensive natural gas development? Energy Policy, 46, 452–459.

[risa13167-bib-0068] Stirling, A. (2007). Risk, precaution and science: Towards a more constructive policy debate. Talking point on the precautionary principle. EMBO Reports, 8(4), 309–315. 17401403 10.1038/sj.embor.7400953 PMC1852772

[risa13167-bib-0069] Stirling, A. (2008). “Opening up” and “closing down”: Power, participation, and pluralism in the social appraisal of technology. Science, Technology & Human Values, 33(2), 262–294.

[risa13167-bib-0070] Strauss, A. L. (1987). Qualitative analysis for social scientists. World, 1, 319.

[risa13167-bib-0071] Taranaki Regional Council (2012). Hydrogeologic risk assessment of hydraulic fracturing for gas recovery in the Taranaki region. Stratford, New Zealand: Taranaki Regional Council.

[risa13167-bib-0072] The Royal Society & Royal Academy of Engineering . (2012). *Shale gas extraction in the UK: A review of hydraulic fracturing* . 10.1016/j.petrol.2013.04.023

[risa13167-bib-0073] Todd Energy . (2012). *Hydraulic fracturing: Submission to the Parliamentary Commissioner for the Environment* .

[risa13167-bib-0074] Upham, P. , Lis, A. , Riesch, H. , & Stankiewicz, P. (2015). Theorising social representations in socio‐technical transitions with the case of shale gas. Environmental Innovation and Societal Transitions, 16, 120–141.

[risa13167-bib-0075] Vasi, I. B. , Walker, E. T. , Johnson, J. S. , & Tan, H. F. (2015). “No fracking way!” Documentary film, discursive opportunity, and local opposition against hydraulic fracturing in the United States, 2010 to 2013. American Sociological Review, 80(5), 934–959.

[risa13167-bib-0076] Visschers, V. H. M. , & Siegrist, M. (2013). How a nuclear power plant accident influences acceptance of nuclear power: Results of a longitudinal study before and after the Fukushima disaster. Risk Analysis, 33(2), 333–347. 22762151 10.1111/j.1539-6924.2012.01861.x

[risa13167-bib-0077] Vorkinn, M. , & Riese, H. (2001). Environmental concern in a local context: The significance of place attachment. Environment and Behavior, 33(2), 249–263.

[risa13167-bib-0078] Walport, M. , & Craig, C. (2014). *Innovation: Managing risk—Not avoiding it* . https://www.gov.uk/government/uploads/system/uploads/attachment_data/file/381905/14-1190a-innovation-managing-risk-report.pdf

[risa13167-bib-0079] Whitmarsh, L. , Nash, N. , Upham, P. , Lloyd, A. , Verdon, J. P. , & Kendall, J. M. (2015). UK public perceptions of shale gas hydraulic fracturing: The role of audience, message and contextual factors on risk perceptions and policy support. Applied Energy, 160, 419–430.

[risa13167-bib-0080] Williams, L. , Macnaghten, P. , Davies, R. , & Curtis, S. (2015). Framing “fracking”: Exploring public perceptions of hydraulic fracturing in the United Kingdom. Public Understanding of Science, 26(1), 89–104. 10.1177/0963662515595159 PMC5207300 26170264

[risa13167-bib-0081] Worksafe (2015). Workplace fatalities by industry. Retrieved: December 10, 2015. https://worksafe.govt.nz/data-and-research/ws-data/fatalities/

[risa13167-bib-0082] Wynne, B. (1992). Uncertainty and environmental learning. Reconceiving science and policy in the preventive paradigm. Global Environmental Change, 2(2), 111–127.

