# Supplementary material for: Lightning Rods, Earthquakes, and Regional Identities: Towards a Multi‐Scale Framework of Assessing Fracking Risk Perception
Source: Risk Anal. 2018 Aug 15;39(2):473–87. doi: 10.1111/risa.13167 (PMC7379633; doi:10.1111/risa.13167)
Supplement: Supplementary file 1 — Supplementary Material [file RISA-39-473-s001.docx]

# Supplementary Material

### 1.1 Interviewee classification

Interviewees were classified into a range of stakeholder types and regional geographies

| **Stakeholder type** | **n=** | **Regional geography** | **n=** |
| --- | --- | --- | --- |
| Local government | 6 | Taranaki | 7 |
| Activist | 4 | Wellington | 6 |
| Expert | 5 | Hawkes Bay | 5 |
| Industry | 4 | Christchurch | 3 |
| Expert Advisory Body | 4 | Auckland | 2 |
| National government | 2 | Waikato | 1 |
|  |  | Otago | 1 |
| **Total** | **25** |  | **25** |

*Table I – Classification of interviews by stakeholder type and regional geography.*

### 1.2 Interview questions

**Opener:**

1. Please tell me about your role / job and how this has resulted in your involvement/interest in hydraulic fracturing operations in New Zealand
   1. Please give me a specific example where you have had personal involvement in a decision that concerns hydraulic fracturing operations
   2. What is your background in terms of education and experience?
2. Please could you give me an overview of the situation concerning hydraulic fracturing firstly in New Zealand and then in (insert current location) specifically?
   1. What do you believe caused the hydraulic fracturing debate to ‘take off’ in New Zealand?
   2. Who do you see as the public groups / identifiable groupings in New Zealand in relation to the hydraulic fracturing agenda?

**Hydraulic Fracturing Perceptions:**

1. Within your professional environment (people you work with daily), how would you describe the perception of risks and opportunities associated with hydraulic fracturing?
   1. What are the key influences on these perceptions? (for example, do engineering standards or quantitative risk analysis play an important role?)
   2. What do you see as the greatest risk associated with hydraulic fracturing?
2. What do you interpret to be the general consensus (or not) amongst the New Zealand public concerning the risks and opportunities surrounding hydraulic fracturing?
   1. Do you think the perception (insert current location) reflects that of the country as a whole?
   2. Do you believe there is an appreciation of the opportunities associated with hydraulic fracturing amongst the New Zealand public?
   3. Within New Zealand, are there certain places where risk perceptions would differ significantly to that of the country as a whole and why?
3. To what extent do you feel that professional and public perceptions of hydraulic fracturing align?
   1. Why do you think a mismatch exists between the various public and professional groups?
   2. Given the objective safety of the technique, why do you think it has become such a controversial debate?
   3. OR If the perceptions are relatively similar, why is there such controversy surrounding hydraulic fracturing?
4. Based on your personal experience and observation of the country as a whole, how have perceptions of hydraulic fracturing changed over time (ie: over the last 25yrs or since 2011)?
   1. Have any new lines of opposition or advocacy emerged/received increasing attention over this period?
   2. Do you anticipate a trend emerging in the future (in, say, the next 10 years)?
5. What significant cultural factors influence perception of hydraulic fracturing and resource extraction more generally?
   1. What do you believe to be the defining features of the national culture of New Zealand (industrial, organizational etc…)
   2. Do you feel that New Zealand has a culture that is shaped or influenced by familiarity with natural disasters such as earthquakes, storms and floods?
   3. Do you think that this results in certain arguments in the hydraulic fracturing debate being emphasised more than others?
   4. Could you tell me about how you believe this plays into the way people perceive other types of risk?
6. Can you recall any significant events that have played into the hydraulic fracturing debate? (for example, one might talk about the Chernobyl disaster in relation to nuclear power)
   1. Why do you think this event had such an impact? (Why was it discussed in relation to hydraulic fracturing specifically?)
   2. Are there any international events that have reverberated in the fracturing debate in New Zealand

**Communicating hydraulic fracturing:**

1. Please could you tell me about a time when you have communicated the risks and opportunities associated with hydraulic fracturing, whether to a friend, a client or the public.
   1. How would you change your approach for another group, if at all (for example, general public vs addressing a group of scientists)? For example, would you use any different language?
2. To what extent do you believe the general public have a good understanding of the process of hydraulic fracturing itself?
   1. Who has a responsibility to ensure that the public have a comprehensive understanding of the science that underpins hydraulic fracturing?
3. What sort of impact do you believe the media have on the way people perceive risk?
   1. How do you utilise the media to communicate the risks and opportunities surrounding hydraulic fracturing?
   2. Do you think the global media play a significant role?
4. Have you had any interaction with pressure groups in opposition to hydraulic fracturing? Please tell me a bit about your experience.
   1. To what extent to you believe these groups are able to influence public opinion?
   2. Which arguments do they use to oppose hydraulic fracturing?
   3. What sort of relationships do you seek to maintain with these groups, and how do you achieve this?
5. Which risks become more prevalent at each of the stages in the hydraulic fracturing operations?
   1. In your experience, at which stage do you experience the most public opposition?
   2. Why do you think this is and how do you manage it?

**Managing hydraulic fracturing:**

1. How has your organisation pursued the ‘social license’ to operate / how do you believe a social license to operate should be gained?
   1. How do you ensure credibility?
   2. How do you build trust?
   3. To what extent do you feel that devolution of power to local councils promotes more progressive dialogue with the general public?
2. What do you see as the biggest challenge to more widespread application of hydraulic fracturing across New Zealand?
   1. How do you see the regulation surrounding hydraulic fracturing developing in the next 10 years?
   2. What would you see change in the approach to managing the public attitudes and engagement with the hydraulic fracturing agenda?

**New Zealand in a global perspective:**

1. Have you been involved in regulation / industry surrounding hydraulic fracturing in other countries?
   1. Please tell me a bit about it OR What is your impression of hydraulic fracturing operations elsewhere?
   2. How does it compare to your experience in New Zealand?
   3. To what extent do you feel that New Zealand’s approach to managing risk perception surrounding hydraulic fracturing differs from other countries?
2. To what extent do you think that perceptions in New Zealand are influenced by or exert influence on other countries’ perceptions of hydraulic fracturing?
   1. Do you think that recent decisions in the UK for example resonate in the policy space in New Zealand?
   2. When discussing hydraulic fracturing in a policy context, are certain foreign policies or events commonly referenced?
   3. What wider global contextual factors do you thing have influenced opinion on hydraulic fracturing? (eg: debates surrounding climate change, energy security or resource use ore generally).

**Finish:**

Is there anything else you would like to tell me?

Is there anyone else that you would recommend that I speak to?

### 1.3 Hierarchical coding and quantitative analysis

Quantitative analysis was performed on coded interviews. Given greater time and resources, individual bias would be minimised through triangulation by asking colleagues to repeat this method. The hierarchical thematic coding approach involved collating the number of times each risk was mentioned in relation to hydraulic fracturing into a spreadsheet for graphical analysis. This method is limited by the subjectivity inherent to categorising complex expressions of risk perception into relatively simple categories such as ‘water contamination’, ‘chemicals’ and ‘seismicity’. In some instances, specific codes were collated into more general categories such as ‘local impacts’. The hierarchical coding schema is presented below.

FRACKING AS RISK (environmental)

-chemicals in the fracking fluid

-contamination of aquifers

-air pollution

-surface water contamination

-contamination of soil

-harm to Papatuanuku

-seismicity

-volumes of water used

-climate change

-well-integrity

-cumulative effects

-wastes

-oil and gas more generally

-radioactivity

FRACKING AS RISK (societal)

-access to land

-local impact on neighbours (social)

-resource consents / regulation

-council capacity and expertise

-don’t trust big business (or cowboys!)

FRACKING AS RISK (identity)
-regional identity

-national identity

-Maori worldview/beliefs/values

FRACKING AS OPPORTUNITY

-jobs

-economic growth

-energy security

-community benefits

-transition fuel argument

DREAD RISK

Uncontrollable / controllable

Dread / Not dread

Global catastrophic / not global catastrophic

Consequences fatal / consequences not fatal

Not equitable / equitable

Catastrophic / individual

High risk to future / low risk to future generations

Not easily reduced / easily reduced

Risk increasing / risk decreasing

Involuntary / voluntary

UNKNOWN RISK

Not observable / observable

Unknown to those exposed / known to those exposed

Effect delayed / effect immediate

New risk / old risk

Risks unknown to science / risks known to science

AFFECT

-language

-imagery

CATASTROPHIC EVENTS

-Christchurch earthquake

-Macondo oil spill

-Rena oil spill

-Pike River mine disaster

MEDIA EVENTS

-*Gasland*

-Promised Land

-Australia documentary (Frackman / Gas Rush)

-national media influence on perceptions

-international responses to fracking

CONSEQUENCES FOR POLICY

-lay understandings of technical knowledge/ expert communication of fracking risk

-PCE Report

-trust

-competency (ie: do the council have the expertise)

-information void

-regional disparity (policy must account for this)

-social license

-if it’s well-regulated, it’ll be fine

### 1.4 Newspaper article automated context analysis

An automated content analysis was performed in Atlasti on the 454 print newspaper articles with mention of the term ‘fracking’. Words that comprised 0.01% or less of the total count were discounted from further analysis, reducing the count from 16208 to 831. All non-specific terms were then excluded, this narrowed the total number of words down to 224. From the remaining list of specific terms, words that could be associated with risks of hydraulic fracturing operations were filtered out and presented.
